# Radiotherapy dose–volume parameters predict facial lymphedema after concurrent chemoradiation for nasopharyngeal carcinoma

**DOI:** 10.1186/s13014-021-01901-7

**Published:** 2021-09-06

**Authors:** Donghyun Kim, Jiho Nam, Wontaek Kim, Dahl Park, Jihyeon Joo, Hosang Jeon, Yongkan Ki

**Affiliations:** 1 Department of Radiation Oncology, Biomedical Research Institute, Pusan National University Hospital and Pusan National University School of Medicine, Busan, Republic of Korea; 2 Department of Radiation Oncology, Pusan National University Yangsan Hospital and Pusan National University School of Medicine, Yangsan, 50612 Republic of Korea

**Keywords:** Lymphedema, Dosimetric predictors, Nasopharyngeal carcinoma, Concurrent chemoradiation

## Abstract

**Background:**

To investigate risk factors for developing radiation-associated facial lymphedema (FL) in nasopharyngeal carcinoma (NPC) patients after concurrent chemoradiation (CCRT).

**Methods:**

Clinical data from 87 patients who underwent definitive CCRT for NPC in 2010–2018 was retrospectively evaluated. FL severity was graded using MD Anderson Cancer Center head and neck lymphedema rating scale. Logistic regression analysis was used to examine the factors associated with the presence of moderate/severe FL (grade ≥ 2).

**Results:**

At a median follow-up of 34 months (range, 18–96), 26/87 (29.9%) patients experienced grade ≥ 2 FL. A majority (84.6%) was experienced grade ≥ 2 FL 3–6 months after CCRT. Mean dose to the level IV, level I-VII neck node and N stage were significantly correlated with grade ≥ 2 FL at univariate analysis. At multivariate analysis, mean dose of level IV neck node (hazard ratio [HR], 1.238; 95% confidence interval [CI] = 1.084–1.414; *p* = 0.002) and level I-VII neck node (HR, 1.384; 95% CI = 1.121–1.708; *p* = 0.003) were independent predictors. Receiver Operating Characteristics (ROC) curve analysis showed that cut-off value of mean level IV neck node dose was 58.7 Gy (area under the curve [AUC] = 0.726; 95% CI = 0.614–0.839, *p* = 0.001) and mean level I-VII neck node dose was 58.6 Gy (AUC = 0.720; 95% CI = 0.614–0.826, *p* = 0.001) for grade ≥ 2 FL.

**Conclusions:**

Keeping mean dose to the level IV and level I-VII below 58.7 Gy and 58.6 Gy may reduce the likelihood of moderate/severe FL after CCRT for NPC.

## Background

Intensity modulated radiotherapy (IMRT) with concurrent chemotherapy is associated with improved disease control for advanced nasopharyngeal carcinoma (NPC). However, there are concerns about the treatment-related toxicities caused by the combination of concurrent chemotherapy and radiotherapy (RT). Severe late toxicities can be life-threatening or significantly impair the patient’s quality of life (QoL) and functional status [1]. Thus, functional outcomes have great importance in true therapeutic success.

Lymphedema (LE) is one of the under-reported but common side effects after RT for head and neck cancer (HNC). A prevalence study of 81 patients at a single institution found 75.3% of patients with HNC presented head and neck LE [2]. Treatment for HNC may disrupt lymphatic structures and damage surrounding soft tissues, leading to increased accumulation of protein rich fluid in interstitial spaces. The retention of lymphatic fluid activates inflammatory responses and eventually leads to skin and subcutaneous soft tissue fibrosis, which can cause decreased neck range of motion [3–5]. Effects of gravity will influence natural edema pooling mechanisms, leading to the submandibular region being the main affected area in facial lymphedema (FL) [6]. Radiation-associated FL has detrimental effects on patient’s QoL because it worsens the appearance. Unlike limb lymphedema, which can be covered by clothing, it is unable to be hidden. Therefore, the potential clinical impact of FL is profound.

It is particularly important to discover risk factors contributing to RT-associated FL in order to identify preventable causes. Currently, an understanding of the RT dose-volume parameters causing FL is limited. We hypothesized that the probability of FL depends on radiation dose and volume delivered to neck lymphatic structures. The purpose of this study was to investigate the relationships between FL and radiation dose to neck lymphatics in patients with NPC after concurrent chemoradiation (CCRT).

## Methods

### Patients

Patients with biopsy-proven NPC and treated with definitive CCRT between January 2010 and December 2018 at our hospital were considered for the present retrospective study. Eligibility criteria were: (1) No surgical operations in the head and neck region during follow-up period to exclude the effect of surgery on FL, (2) IMRT as a radiation treatment modality, (3) Pretreatment head or neck LE grade ≤ 1b, and (4) minimum follow-up of 18 months.

### Treatment

Treatment planning was conducted using TomoTherapy planning system in all patients (Accuray Precision version 1.1.1.1: Accuray Inc., Madison, WI). IMRT was delivered through TomoTherapy (Accuray Inc., Sunnyvale, CA, USA). Definitive RT was delivered, in conjunction with weekly intravenous cisplatin (40 mg/m^2^) as a radiosensitizing agent (median 6 cycles, range 3–7). The prescribed dose to the gross tumor volume and macroscopically enlarged lymph node was 66–70 Gy/2.0 Gy fraction; prophylactic level doses to nodes was 50–60 Gy/2.0 Gy fraction according to the subclinical disease risk. After completion of CCRT, 40 patients underwent consolidation chemotherapy (CCT) every 3 weeks for a total of three cycles according to the medical oncologist's preference. Docetaxel 70 mg/m^2^ diluted in 300 ml of 5% dextrose water was administered over 2 h followed by cisplatin 75 mg/m^2^ diluted in 200 ml of normal saline administered over 90 min.

### Facial lymphedema assessment

FL severity was routinely graded at each visit for all patients according to the MD Anderson Cancer Center (MDACC) head and neck LE rating scale [4]. The cut-off level for clinically significant FL was taken as grade ≥ 2. The maximum FL grade during the follow-up period was used for scoring. Time to endpoint was assessed from the date of treatment end to the time of the first observation of grade ≥ 2 FL. We dichotomized groups with FL cutoff grade ≥ 2 as moderate/severe FL because grade ≥ 2 FL is irreversible and has lower QoL.

### Dosimetric data

Planning computed tomography (CT) Digital Imaging and Communications in Medicine (DICOM) files and associated dosimetric data were exported to a commercially available deformable image registration and segmentation software program (Mim Maestro, MIM software Inc., Cleveland, OH, USA). The neck node levels (from I to VII) were individually delineated on each planning CT by one observer consistent with a previously published guideline [7], and subsequently reviewed by two trained radiation oncologists. We extracted the mean dose (D_mean_) of neck node levels and neck node level-specific dose-volume histograms (DVH) with a dose bin size of 0.1 Gy for further analysis.

### Statistical analysis

The association between the development of grade ≥ 2 FL and clinical variables (age, gender, smoking [smoking history of at least 10 pack years], alcohol use [drinking alcoholic beverage during the follow-up period], hypertension, diabetes mellitus, body mass index [BMI] ≥ 30, T stage, N stage, CCT and mean dose of neck node levels) were evaluated by using binary logistic regression analysis. Covariates with values of *p* < 0.1 at univariate analysis were entered into a Cox proportional multivariate analysis. A backward selection procedure based on the likelihood ratio test was used to select variables. All tests were two-tailed and conducted at a 5% significance level (*p* < 0.05). Receiver operating characteristic (ROC) curve analysis was used to determine areas under the curve (AUC) to estimate the accuracy and predictive value of dosimetric parameters for grade ≥ 2 FL. All statistical analysis was performed with SPSS software, version 18.0 (SPSS Inc., Chicago, IL, USA).

## Results

### Patient characteristics

Among the 87 patients eligible for analysis, 62 were male (71.3%) and the median age was 54 (range 21–77) years. One third of participants reported a smoking history, and 43.7% of participants reported alcohol consumption. Advanced stage disease (III–IV) was present in 75.9% of all participants. All patients were treated comprehensively on both sides of the neck. Median total dose was 70 (range 62–72) Gy delivered using standard fractionation. Patient and treatment characteristics are summarized in Table 1.

**Table 1:** Patient and tumor characteristics by facial lymphedema status after CCRT

	All patients N = 87 (%)	Facial lymphedema (grade ≤ 1B) N = 61	Facial lymphedema (grade ≥ 2) N = 26	Univariate analysis *P* value
Age, median (range)	54 (21–77)	53 (32–77)	59 (21–73)	0.694
≥ 60	26 (29.9)	19	7	
< 60	61 (70.1)	42	19	
Gender				0.807
Male	62 (71.3)	43	19	
Female	25 (28.7)	18	7	
Smoking status				0.868
Yes	29 (33.3)	20	9	
No	58 (66.7)	41	17	
Alcohol use				0.523
Yes	38 (43.7)	28	10	
No	49 (56.3)	33	16	
Hypertension				0.587
Yes	20 (23.0)	15	5	
No	67 (77.0)	46	21	
Diabetes mellitus				0.617
Yes	11 (12.6)	7	4	
No	76 (87.4)	54	22	
Body mass index				0.195
≥ 30	22 (25.3)	13	9	
< 30	65 (74.7)	48	17	
T stage**				0.498
1–2	45 (51.7)	33	12	
3–4	42 (48.3)	28	14	
N stage**				0.099*
0–1	42 (48.3)	33	9	
2–3	45 (51.7)	28	17	
Consolidation chemotherapy				0.155
Yes	40 (46.0)	25	15	
No	47 (54.0)	36	11	
Baseline facial lymphedema grade***				0.752
0	79 (90.8)	55	24	
1a	8 (9.2)	6	2	
Mean dose (Gy) to neck node (standard deviation)				
Level I	59.0 (5.1)	58.5 (6.1)	60.1 (2.3)	0.477
Level II	66.6 (2.3)	66.2 (2.2)	67.6 (2.8)	0.613
Level III	62.4 (3.2)	61.3 (2.5)	65.0 (3.7)	0.339
Level IV	57.2 (3.9)	55.2 (3.9)	60.9 (3.6)	0.001*
Level V	59.9 (3.4)	59.1 (3.6)	61.7 (2.3)	0.226
Level VI	44.7 (6.3)	44.5 (6.6)	45.1 (7.0)	0.81
Level VII	67.3 (2.9)	68.0 (1.9)	65.8 (4.7)	0.127
Level I–VII	60.5 (2.9)	59.8 (3.0)	62.1 (2.1)	0.002*

*Statistically significant *P* value < 0.1

**TNM classification per AJCC staging 8th edition

***MD Anderson Cancer Center head and neck LE rating scale

### Facial lymphedema classification

Distribution of baseline FL was as follows: 79 patients (90.8%) grade 0 and 8 (9.2%) grade 1a. At a median follow-up of 34 months (range, 18–96), 26/87 (29.9%) patients experienced grade ≥ 2 FL. A majority (84.6%) experienced grade ≥ 2 FL 3–6 months after CCRT with all events occurring within 15 months. The median duration of grade ≥ 2 FL was 5.0 months; 18 patients recovered within 6 months and grade ≥ 2 FL persisted in 5 patients until last follow-up date. There was no grade 3 FL during follow-up period. The sites most frequently involved were the submental (92.3%) and submandibular (76.9%) region.

### Correlates of facial lymphedema (Grade ≥ 2) with clinical variables

**Fig. 1:**
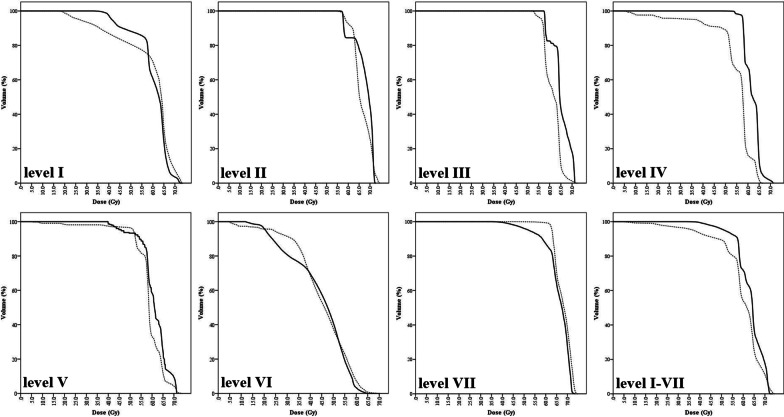
Averaged dose–volume histograms for patients with (solid) and without (dashed) facial lymphedema (grade ≥ 2)

For all tested neck node levels, mean dose to level IV and I-VII were uniformly higher for patients with grade ≥ 2 FL (Table 1) and included in the multivariate model. Mean level IV and level I-VII neck node dose was 57.2 Gy (FL grade < 2, 55.2 Gy vs. FL grade ≥ 2, 60.9 Gy; *p* = 0.001) and 60.5 Gy (FL grade < 2, 59.8 Gy vs. FL grade ≥ 2, 62.1 Gy; *p* = 0.002), respectively. Figure 1 shows averaged cumulative DVHs for patients with and without grade ≥ 2 FL. DVHs graphically demonstrated that patients with grade ≥ 2 FL had higher dose delivery with some variability of magnitude across neck node levels.

Results of univariate analysis for clinical variables are reported in Table 1. The nodal stage was associated with an increased likelihood of grade ≥ 2 FL (*p* = 0.099), while the remaining patient, tumor and treatment-related factors failed to demonstrate an association with grade ≥ 2 FL. Multivariate Cox proportional analysis model including the N stage and mean neck node dose indicated mean level IV dose (hazard ratio [HR], 1.238; 95% confidence interval [CI], 1.084–1.414; *p* = 0.002) and mean level I-VII dose (HR, 1.384; 95% CI, 1.121–1.708; *p* = 0.003) as the independent predictors of grade ≥ 2 FL (Table 2).

**Table 2:** Multivariate analysis of risk factors associated with grade ≥ 2 radiation-associated facial lymphedema after CCRT

Clinical and dosimetric characteristic	HR (95% CI)	*P* value*
N stage (0–1 vs. 2–3)	1.392 (0.744–2.357)	0.270
Mean dose (Gy) to level IV	1.238 (1.084–1.414)	0.002**
Mean dose (Gy) to level I–VII	1.384 (1.121–1.708)	0.003**

*HR* hazard ratio, *CI* confidence interval

*P value were calculated by backward Cox hazard model

**Statistically significant *P* value < 0.05

### Threshold neck node dose for facial lymphedema (Grade ≥ 2)

**Fig. 2:**
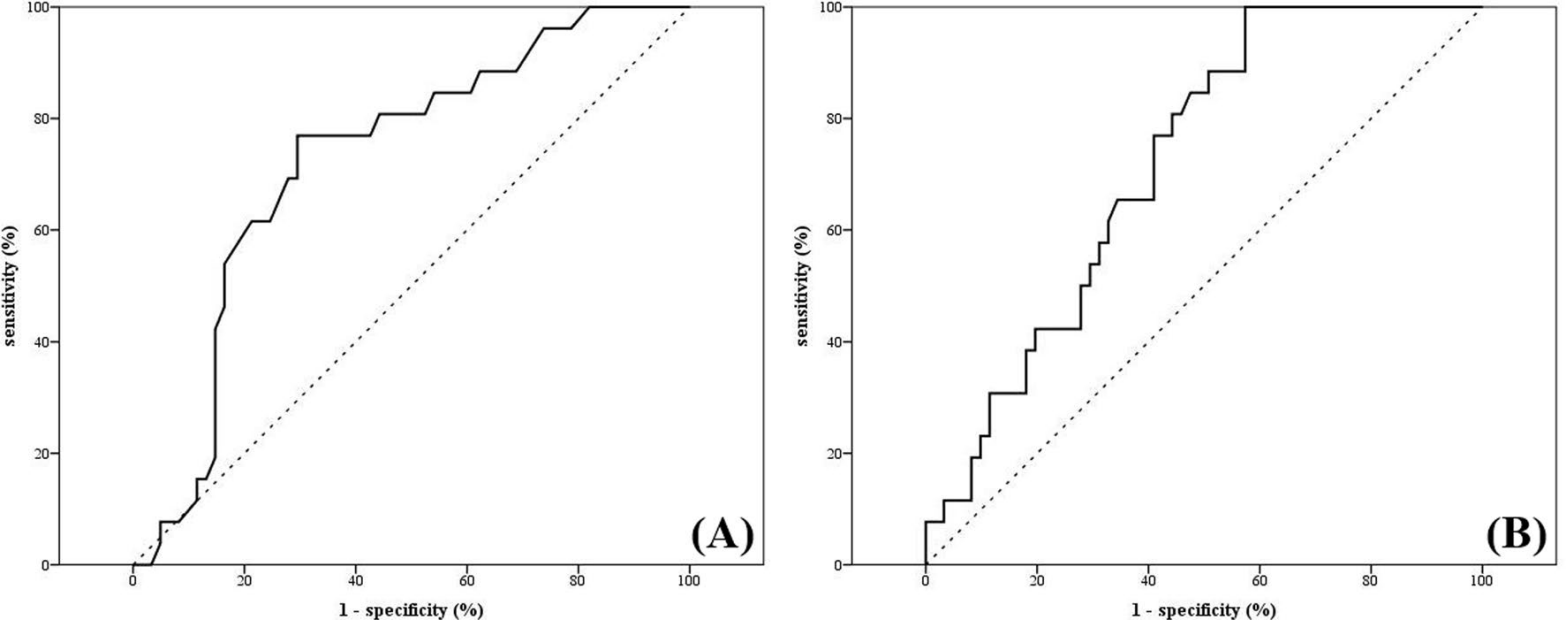
Receiver operating characteristics (ROC) curves of grade ≥ 2 facial lymphedema according to mean level IV (**a**) and level I-VII (**b**) neck node dose in all patients (n = 87). Optimal cut-off mean dose of level IV neck node was 58.7 Gy (AUC: 0.726; 95% CI = 0.614–0.839, *p* = 0.001) and level I-VII neck node was 58.6 Gy (AUC: 0.720; 95% CI = 0.614–0.826, *p* = 0.001), respectively

The calculation of AUC of ROCs showed that the probability of grade ≥ 2 FL increases with higher D_mean_ for level IV and level I–VII. Specifically, optimal cut-off mean dose of level IV neck node was 58.7 Gy (AUC: 0.726; 95% CI = 0.614–0.839, p = 0.001) and level I–VII neck node was 58.6 Gy (AUC: 0.720; 95% CI = 0.614–0.826, p = 0.001) (Fig. 2).

## Discussion

The present study shows that the risk of radiation-associated moderate/severe FL is correlated with dosimetric variables. Among them, mean dose to level IV and level I–VII neck node are the best predictors. This report identified the mean dose of whole neck node and lower jugular node are most strongly contribute to FL. Since bilateral upper and middle jugular node is mostly exposed to high doses and have a small dosimetric difference in RT for NPC, lower jugular node seems to have a more pronounced difference. The authors therefore assume that mean dose of level I–VII is more appropriate for use in the prediction of moderate/severe FL after CCRT of most HNC. We also proposed dose constraints derived for these structures in the present analysis.

It has been reported that FL presents in more than 70% of patients after HNC treatment and primary tumor site in the pharynx, combined treatment modality, high RT dose, and RT duration were statistically significantly associated with presence of LE [8, 9]. We did not find that any of the patient, tumor, or treatment-related factor was associated with FL in our study population. Although N stage (*P* = 0.099) demonstrated a predictive potential for grade ≥ 2 FL in the univariate analysis for clinical factors, this association did not maintain in multivariate models once dosimetric variables were included. Nodal stage may be considered a surrogate for the extent of normal tissue damage secondary to treatment; thus, we expected that increasing stage would be associated with increased incidence of grade ≥ 2 FL. Dosimetric factors may play a more profound role in RT-associated grade ≥ 2 FL, negating any potential effect of tumor related factors in this study population.

Our results show that sparing of part of neck node from radiation exposure may result in a significant reduction of the development of grade ≥ 2 FL. This point is important and confirms that unnecessary irradiation of the both sides of neck should be avoided. Omitting contralateral neck radiation significantly improves patient-reported QoL. Previous studies have shown that elective ipsilateral radiation results in low rates of contralateral regional recurrence in patients with well-lateralized tonsillar cancer [10]. There is growing evidence that the incidence of contralateral neck recurrence in properly-selected HNC is very low [11, 12] and we assume that bilateral elective neck irradiation (ENI) is an overtreatment in the majority of patients with well-lateralized HNC.

The ENI dose of 44–64 Gy to the clinically uninvolved lymphatics has usually been recommended in most HNC types [13]. A prospective study in HNC patients reported that lower ENI dose (40 Gy vs. 50 Gy) was not inferior with respects to locoregional control and survival outcome [14]. There is now great interest in investigating the reduction of radiation therapy dose prescription for elective nodal areas to improve the therapeutic ratio (maintain excellent cancer control and decrease toxicity) in human papilloma virus-associated oropharyngeal carcinoma [15]. A phase II study in HNC patients also revealed that the lower elective dose of 36 Gy improved the patient-reported QoL score [16].

## Conclusions

We provide evidence that moderate/severe FL is strictly correlated with the mean dose of level IV and level I-VII neck node. To minimize the risk of grade ≥ 2 FL, mean neck node level IV and level I-VII dose should be kept as low as possible, more specifically < 58.7 Gy and < 58.6 Gy, respectively. Head and neck oncologists need to conduct lymphedema assessment as a component of routine clinical examination and consider rehabilitation consultations, especially for patients receiving high mean dose to neck nodal region.

## Data Availability

All data analyzed during this study are available from the corresponding author on reasonable request.

## Abbreviations

AUC: Areas under the curve
BMI: Body mass index
CCRT: Concurrent chemoradiation
CCT: Consolidation chemotherapy
CI: Confidence interval
CT: Computed tomography
DICOM: Digital Imaging and Communications in Medicine
DVH: Dose-Volume Histograms
ENI: Elective neck irradiation
FL: Facial lymphedema
HNC: Head and neck cancer
HR: Hazard ratio
IMRT: Intensity modulated radiotherapy
LE: Lymphedema
MDACC: MD Anderson Cancer Center
NPC: Nasopharyngeal carcinoma
QoL: Quality of life
ROC: Receiver operating characteristic
RT: Radiotherapy
